# Skeletal Development of Mice Lacking Bone Sialoprotein (BSP) - Impairment of Long Bone Growth and Progressive Establishment of High Trabecular Bone Mass

**DOI:** 10.1371/journal.pone.0095144

**Published:** 2014-05-09

**Authors:** Wafa Bouleftour, Maya Boudiffa, Ndeye Marième Wade-Gueye, Guénaëlle Bouët, Marco Cardelli, Norbert Laroche, Arnaud Vanden-Bossche, Mireille Thomas, Edith Bonnelye, Jane E. Aubin, Laurence Vico, Marie Hélène Lafage-Proust, Luc Malaval

**Affiliations:** 1 Laboratoire de Biologie du Tissu Osseux and Institut National de la Santé et de la Recherche Médicale - U1059, Université de Lyon - Université Jean Monnet, Saint-Etienne, France; 2 Dept. of Molecular Genetics, University of Toronto, Toronto, Ontario, Canada; 3 Institut National de la Santé et de la Recherche Médicale - U1033, Université de Lyon - Université Claude Bernard, Lyon, France; Institut de Génomique Fonctionnelle de Lyon, France

## Abstract

Adult *Ibsp-*knockout mice (BSP−/−) display shorter stature, lower bone turnover and higher trabecular bone mass than wild type, the latter resulting from impaired bone resorption. Unexpectedly, BSP knockout also affects reproductive behavior, as female mice do not construct a proper "nest" for their offsprings. Multiple crossing experiments nonetheless indicated that the shorter stature and lower weight of BSP−/− mice, since birth and throughout life, as well as their shorter femur and tibia bones are independent of the genotype of the mothers, and thus reflect genetic inheritance. In BSP−/− newborns, µCT analysis revealed a delay in membranous primary ossification, with wider cranial sutures, as well as thinner femoral cortical bone and lower tissue mineral density, reflected in lower expression of bone formation markers. However, trabecular bone volume and osteoclast parameters of long bones do not differ between genotypes. Three weeks after birth, osteoclast number and surface drop in the mutants, concomitant with trabecular bone accumulation. The growth plates present a thinner hypertrophic zone in newborns with lower whole bone expression of IGF-1 and higher IHH in 6 days old BSP−/− mice. At 3 weeks the proliferating zone is thinner and the hypertrophic zone thicker in BSP−/− than in BSP+/+ mice of either sex, maybe reflecting a combination of lower chondrocyte proliferation and impaired cartilage resorption. Six days old BSP−/− mice display lower osteoblast marker expression but higher MEPE and higher osteopontin(Opn)/Runx2 ratio. Serum Opn is higher in mutants at day 6 and in adults. Thus, lack of BSP alters long bone growth and membranous/cortical primary bone formation and mineralization. Endochondral development is however normal in mutant mice and the accumulation of trabecular bone observed in adults develops progressively in the weeks following birth. Compensatory high Opn may allow normal endochondral development in BSP−/− mice, while impairing primary mineralization.

## Introduction

Members of the SIBLING (Small Integrin-Binding Ligand, N-linked Glycoprotein) family [1] are known to play key biological roles in the development, turnover and mineralization of bone and dentin. Among this family, bone sialoprotein (BSP, IBSP, [2]) as well as osteopontin (Opn, SPP1, [3]) are strongly expressed by osteoblasts, osteoclasts and hypertrophic chondrocytes. Incipient expression of BSP, which is particularly abundant in sites of primary bone formation [4] [5] coincides with the initial formation of membranous and endochondral bone, maximal level being reached during the formation of embryonic bone [5]. We previously showed that adult mice (16 week old) with a knockout of the *Ibsp* gene (BSP−/−) are shorter than their wild type counterparts and display a low level of bone remodeling, with both bone formation and mineralization severely impaired *in vivo* and in *in vitro* models [6]. We also showed that BSP−/− mice display lower osteoclast numbers and surfaces *in vivo* [6], and that osteoclast recruitment and activity *in vitro* were impaired in the absence of BSP [7]. Interestingly, and despite their low level of bone remodeling, adult BSP−/− mice present a higher trabecular bone mass than the BSP+/+ [6], which raises questions about the development of the BSP−/− adult phenotype.

In this study, we monitored the skeletal development of BSP−/− and BSP+/+ mice, asking when and how the short size and high trabecular bone of the adult were set up. BSP−/− mothers display an abnormal pup care behavior, which does not impair the growth of wild type and heterozygous mice, showing that the skeletal phenotype of BSP−/− mice is gene-based. We show that BSP−/− mice are born with their shorter stature and that the lack of BSP alters long bone growth, membranous/cortical primary bone formation and mineralization, as well as cartilage and osteoblast gene expression, with low bone IGF-1 and high levels of Opn. However, the endochondral development is normal in mutant mice and the accumulation of trabecular bone observed in the adults develops progressively in the weeks following birth.

## Material and Methods

### Production of BSP−/− mice

BSP Knock-out mice were generated as described in [6]. Briefly, exons II–III of the mouse *Ibsp* gene were replaced by a PGK_neo_ cassette that created a null allele in mouse embryonic stem (ES) cells (R1 passage 8; kindly provided by Dr. Andras Nägy [8]). After selection, positive clones were used to generate chimeric mice which were crossed to albino CD1 outbred females, and a chimeric male with germline transmission of the mutation was used to establish offspring on a 129/CD1 background. BSP−/− mice are viable and fertile; their phenotype has been extensively described elsewhere [6].

### Animal reproduction and genotyping

Wild type and mutant mice were kept as separate lines, except for cross-foster experiments. They were housed under controlled conditions at 23±2°C on a 12-h light/12-h dark cycle, fed with A03 food preparation (SAFE, Avry, France) and given tap water with free access. Whenever required, the offsprings genotyping was performed by PCR (Qiagen, Mannhein,Germany) on genomic DNA Tail isolated with Direct PCR Lysis Reagent (Viagen, Biotech Inc., Los Angeles, Ca, USA) plus proteinase K (Euromedex, France). The PCR reaction was performed with the following primers: BSP-forward = AGGACTAGGGGTCAAACAC, BSP-reverse = AGTAGCGTGGCCGGTACTTA, and PKG_neo_ (RLP 290) = TCGCCTTCTTGACGAGTTCTGAG, in the appropriate PCR conditions. Results were analyzed by running the samples in a 2% agarose (Sigma, France) gel electrophoresis. Expected bands were at 481 bp for BSP+/+, 550 bp for BSP−/− and both for BSP+/− samples.

### Sampling procedures

Newborn mice were killed by hypothermia, and fixed in 100% EtOH for 24 h. Three, 10 and 16 week old mice were killed by cervical dislocation and the femurs and tibias dissected out and fixed in 3.7% paraformaldehyde in PBS. Six days, 35 days and 12 month old animals were killed by decapitation for serum collection and storage at −80°C. For 6 days old mice, blood was collected from 5–6 individuals and pooled in the same tube (N in the results thus refers to the number of blood pools assayed). In some 6 days old mice, long bones were dissected out and processed for QRT-PCR as described below. The procedures for the care and killing of the animals were in accordance with the European Community Standards on the care and use of laboratory animals (Ministère de l′Agriculture, France, Authorization 04827). All animal experiments were approved by the "Comité du Bien Etre Animal" of the medical faculty experimentation platform (PLEXAN, Université Jean Monnet, Saint-Etienne, France).

### Morphological and morphometric assessment

Fixed newborn mice were dissected, skinned, eviscerated and dehydrated in 100% acetone for 24 h. The samples were then processed for whole mount skeletal preparation according to established methods [9]. Alcian blue was used to stain cartilage and Alizarin red was used to stain calcified tissues.

To check whether the altered behavior of BSP−/− mothers had impact on their bone phenotype, multiple crossing experiments were performed. We first crossed BSP+/+ with BSP−/− mice to generate heterozygous progeny. Then, female BSP−/− mice were crossed with heterozygous BSP+/− male mice, giving a mixed progeny of BSP−/− and BSP+/− pups. Finally, we crossed all heterozygous mice to obtain a Mendelian mix of BSP+/+, −/− and +/− progeny. In all configurations, we compared length and weight growth curves between 8 and 35 days, as well as femur length at 40 days in pups of different genotypes raised by BSP+/+, +/− or −/− mothers. To do this, mice were tagged for identification at day 7 after birth, and then weighted every other day with a 0.1 g precision scale. Their length was measured (nose to tail insertion) with an electronic digital caliper. At day 40, mice of all genotypes were killed and the femurs and tibias collected (see above) were measured with a caliper. In all cases, we took care to compare mice from litters of similar sizes.

### Tridimensional microtomography (µCT)

Newborn whole skeletons and femurs from 3, 10 and 16 week old BSP+/+ and BSP−/− mice were scanned with a high resolution µCT (Viva CT40, Scanco Medical, Bassersdorf, Switzerland). Newborn mice, were scanned at 45 keV with a 10 µm cubic resolution. For isolated femurs, the secondary spongiosa of trabecular bone was scanned within the metaphysis below the growth plate and the cortical bone was scanned in the diaphysis, at 55 keV with a 10 µm cubic resolution. Three-dimensional reconstructions were generated using the following parameters for newborn whole skeletons: Sigma, 1.2; Support, 2; Threshold, 225. Parameters were; Sigma, 1.2; Support, 2; Threshold, 276 for trabecular bone and Sigma, 1.2; Support, 2; Threshold, 280 for cortical bone of 3 week old femurs, and Sigma, 1.2; Support, 2; Threshold, 245 for trabecular bone and Sigma, 1.2; Support, 2; Threshold, 280 for cortical bone of 10 and 16 weeks femurs. The structural parameters of trabecular bone, bone volume (BV/TV), trabecular thickness (Tb.Th), trabecular number (Tb.N), trabecular separation (Tb.Sp), and structure model index (SMI) were generated from a set of 80 sections, without assuming a constant model, as previously described [10]. Cortical area and thickness (Ct.Th) were calculated by integration of the value on each transverse section of a set of 30 chosen in the midshaft area.

### Bone histology and histomorphometry

Excised and fixed femurs and the lower half of fetuses were dehydrated in acetone and embedded in methylmethacrylate. Longitudinal slices were performed with a Jung model K microtome (Carl Zeiss, Heidelberg, Germany) and used for modified Goldner staining, tartrate-resistant acid phosphatase (TRAP) staining of osteoclasts and Von Kossa staining of calcified tissues. Trabecular bone volume (BV/TV), osteoïd surface (OS/BS), growth plate thickness and hypertrophic zone thickness were measured on Goldner stained sections. Osteoclast number (Oc.N/BS) and osteoclast surface (Oc.S/B.Ar) were measured on TRAP stained sections. ROI for bone parameters were evaluated in the secondary spongiosa of trabecular bone within the metaphysis below the growth plate as shown in the figure.

### RNA extraction and QRT-PCR analysis

Both femur and tibia (in connection and including articulations and growth plates) of 6 days old mice were haversted after death, cleaned of surrounding soft tissue and frozen in liquid nitrogen. Bone samples were crushed with a mixer mill (Sartorius,Göttingen, Germany) using steel balls. RNA was extracted from the bone powder with Tri-Reagent (Sigma), then purified on columns according to the manufacturer's instructions (RNeasy Plus Mini Kit, Qiagen, Hilden, Germany). RNA amounts were assessed with the Ribogreen kit (Invitrogen, Life Technologies, Eugene, OR, USA) and their quality checked with the Experion automated electrophoresis station (BIO-RAD, Hercules, CA, USA). Messenger RNA was reverse-transcribed (iScript cDNA synthesis Kit, Biorad) according to manufacturer's instruction, then 400 ng of cDNA were amplified through QRT-PCR using the SYBR Green I dye (Lightcycler faststart DNA master SYBR green I, Roche, Germany). Expression of the genes of interest was normalized to glyceraldehyde-3-phosphate dehydrogenase (GADPH). The expression of the housekeeping gene did not differ at either age in either genotype. Primer sequences of the transcripts amplified are listed in Table 1.

**Table 1:** PCR primer sequences in 5′-3′ direction.

Gene	Forward	Reverse	Sequence Bank ID
Runx2	ccgggaatgatgagaactac	Tgtctgtgccttcttggttc	NM_009820.4
Osx	atggcgtcctctctgcttg	aaggtcagcgtatggcttct	NM_130458.3
Ocn	ctctgacctcacagatgccaa	ctggtctgatagctcgtcaca	NM_007541.2
DMP1	ttcgagaacttcgctgaggt	ttgtggtatctggcaactgg	NM_016779.2
MEPE	ccccaagagcagcaaaggta	ctccgctgtgacatcccttta	NM_053172.2
Opn	cccggtgaaagtgactgattc	atggctttcattggaattgc	NM_009263.2
PTHrP	ctggttcagcagtggagtgt	cctccgaggtagctctgatt	NM_008970.3
IHH	cgctgcaaggaccgtctgaa	agcggccgaatgctcagact	NM_010544.2
IGF-1	cttcaccagctccaccacag	cagctccggaagcaacactc	NM_010512.4
GAPDH	aaggtcggtgtgaacggatt	attctcggccttgactgtgc	NM_008084

The meaning of the abbreviations is explained in the text.

### ELISA assays

Serum samples were assayed with a commercial ELISA kits for Opn (DuoSet mouse Osteopontin, R&D Systems, Minneapolis, MN, USA) used according to the manufacturer's instructions. Serum was obtained as described in sampling procedures. Each sample was run in duplicate in the assay.

### Statistical Analysis

All results are presented as mean ± SEM. To accommodate for sample size variations according to the experiment (cf. Figure Legends), and N values sometimes too small to establish normality, all data were submitted to Kruskall and Wallis with post-test or Mann-Whitney U test (see Legends), performed with the INSTAT software (version 3.00 for Windows 95, GraphPad Software, San Diego, CA).

## Results

### Developmental effects of the BSP gene knockout

**Figure 1:**
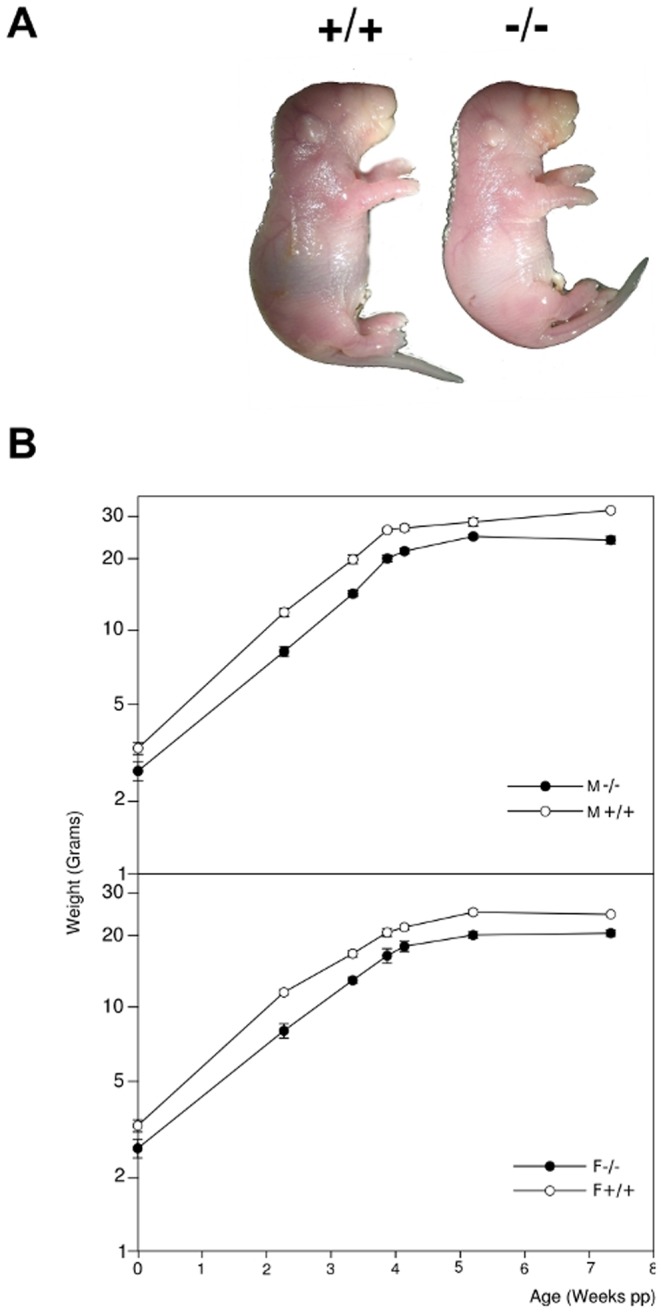
Newborn morphology and growth of BSP+/+ and BSP−/− mice. (A) general aspect of BSP+/+ (+/+) and BSP −/− (−/−) newborn mice. (B) Kinetics of weight gain during the growth of BSP+/+ and BSP−/− mice. M, males; F, females. Data are Mean±SEM of N = 4 to 31 mice. Note the log scale.

BSP−/− mice were significantly smaller than BSP+/+, since birth (Fig. 1A) and up to senescence as shown by weight curves (Fig. 1B) and as previously reported from long bone measurements [6].

### Delayed skeletal mineralization in mice lacking BSP

**Figure 2:**
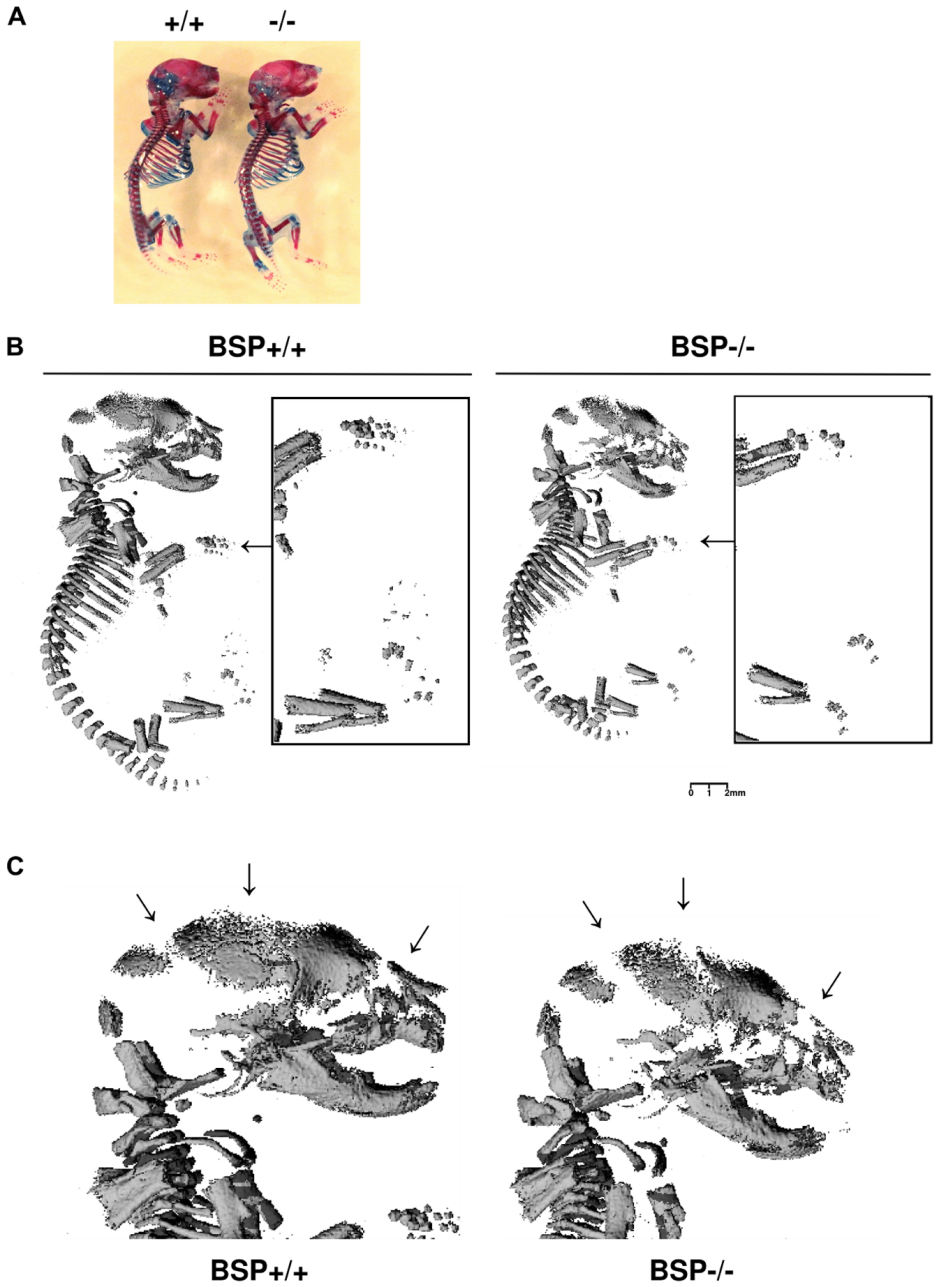
Skeletal development and mineralization of newborn BSP+/+ and BSP−/− mice. (A) Skeletal preparation (alcian blue/alizarin red staining) of BSP+/+ (+/+) and BSP −/− (−/−) E19 mice. (B) 3D µCT reconstruction of whole newborn +/+ and −/− mice, showing undermineralized fingers (arrows and black-bordered detailed pictures), and (C) high magnification of the heads showing the widened sutures (arrows).

General aspect or whole mount skeletal preparations of newborn BSP+/+ and BSP−/− mice revealed no differences in morphology nor endochondral ossification stage (Fig. 2A), nor did histological observations indicate any delay in the appearance of ossification centers (not shown). However, the skeletal elements of newborn BSP−/− mice were under-mineralized respective to BSP+/+, as showed in 3D µCT reconstructions by missing finger bones (Fig. 2B) porous skull elements and widened sutures (Fig. 2C).

### The altered reproductive behavior of BSP−/− mothers does not bear on the expression of the mutant phenotype

**Figure 3:**
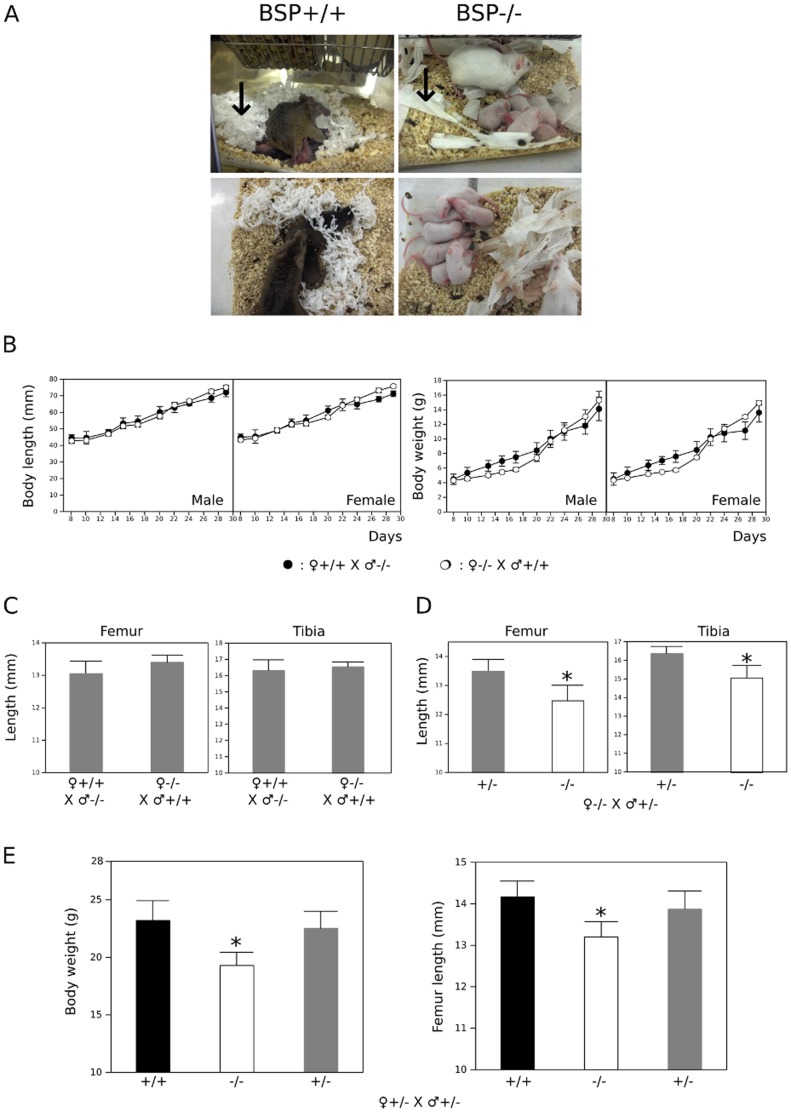
Impact of the lack of BSP on mother's behavior. (A) Pictures of BSP+/+ and BSP−/− mothers with pups; note the well formed "nest" in the wild type mouse cage as opposed to the untouched and spread out pieces of soft paper in the mutant mouse cage (arrows). Note: the 129 sv/CD1 background is outbred, leading to hair color variability. (B) Body length (left) and weight (right) increase in the 30 first days of growing male and female BSP+/− mice from BSP+/+ (○, N = 7 pups) and BSP−/− mothers (•, N = 6 pups). (C) Femur and tibia length of 40 days old BSP+/− mice from either BSP+/+ or BSP−/− mothers. (D) Femur and tibia length of 40 days old BSP+/− (N = 6) and BSP−/− mice (N = 6) from BSP−/− mothers crossed with BSP+/− males. (E) Body weight and femur length of 40 days old BSP+/+ (N = 7), BSP−/− (N = 11) and BSP+/− mice (N = 22) from BSP+/− parents. Data are Mean±SEM; *: p<0.05 vs BSP+/+ and/or BSP+/−, Mann-Whitney U Test or (E) Kuskall-Wallis with post-test.

Observation of weaning wild type and mutant mice revealed that mothers lacking BSP display an aberrant behavior. Pieces of soft paper were put in the cages 5 days before birth to provide the mothers with proper material for nest construction [11]. While BSP+/+ mice built nests for their offsprings by carefully dilacerating the paper material, BSP−/− mothers did not tear the paper apart and hardly gathered the fragments around their pups (Fig. 3A). Although this was not quantified, BSP−/− mothers also spent more time wandering in the cages and less time in contact with the pups than the BSP+/+.

Concerned that altered care for the offsprings might be a confounding factor, we analyzed the effect of the genotype of mothers on the development of the BSP−/− phenotype in the pups and designed cross-fostering experiments. First, we crossed mutant and wild type mice and assessed the growth curve of heterozygous offsprings. As shown in Figure 3B, the mother's genotype did not affect the rate of weight or length accrual during the weaning period. Also, femur and tibia length of 40 days old BSP+/− mice did not differ whether they were raised by +/+ or −/− mothers (Fig. 3C). Second, we crossed BSP−/− females with +/− males and measured femur and tibia length at day 40; as expected and published before [6], we found that the long bones of −/− offsprings were shorter than those of +/− siblings of the same −/− mother (Fig. 3D). Conversely, the crossing of +/− mice gave a mendelian ratio (not shown) of +/+, −/− and +/− offsprings, with significantly lower body weight and femur length only in the BSP−/− (Fig. 3E), reflecting the effect of the mutation and its recessive character, as previously described [6]. Therefore, no mother effect was observed for the skeletal impact of the absence of BSP, which appears to be strictly gene based.

### BSP−/− mice display altered growth plate parameters

**Figure 4:**
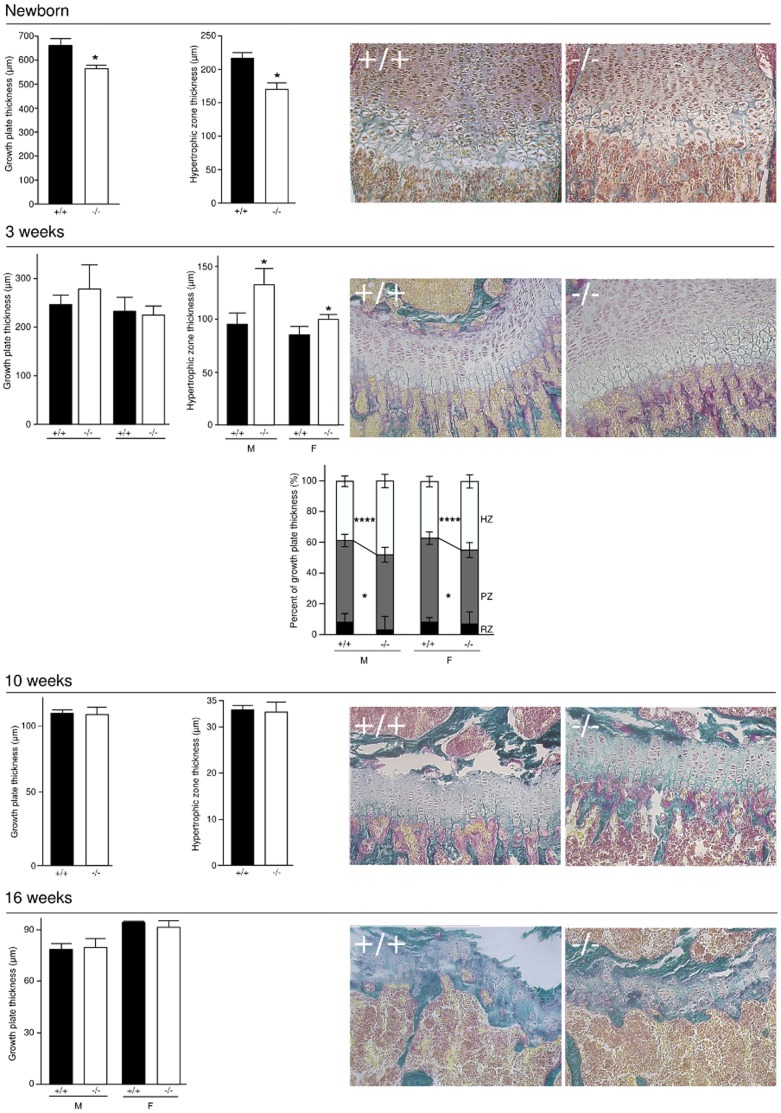
Growth plate kinetics of newborn, 3 week old, 10 and 16 week old BSP+/+ and BSP−/− mice. Data are Mean±SEM of N = 7 to 8 newborn and 4 to 6 adult mice; *:p<0.05, **:p<0.001 vs BSP+/+ Mann-Whitney U Test. Micrographs of the growth plate in the distal femur of BSP+/+ and BSP−/− newborn and 3, 10 and 16 week old mice.

In newborn BSP−/− mice, growth plate thickness was significantly lower than in BSP+/+, and the hypertrophic zone was thinner (Fig. 4). At 3 weeks of age, growth plate thickness was similar in both genotypes, but with a thicker hypertrophic zone and a thinner proliferating zone in both sexes. No difference was observed between the two genotypes in the growth plates of 10 week old femurs, nor at 16 weeks when growth activity was residual and the plates were starting to mineralize (Fig. 4).

### Newborn BSP−/− mice display thinner, hypo-mineralized cortical bone

**Figure 5:**
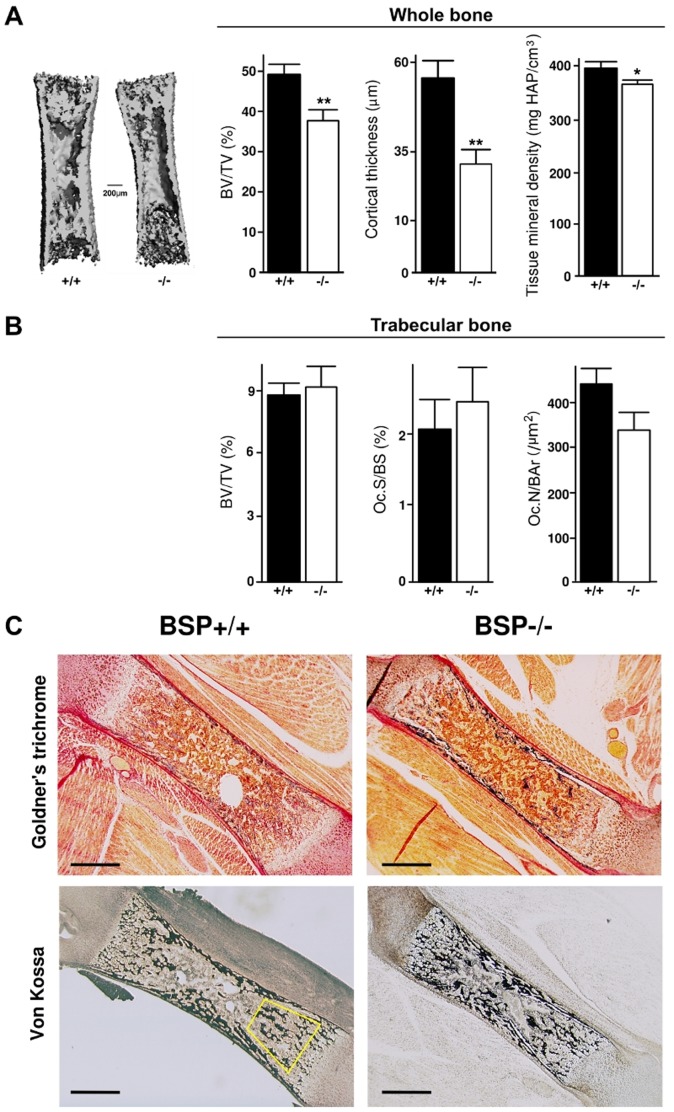
Morphology and morphometry of the long bones in newborn BSP+/+ and BSP−/− mice. (A) 3D µCT reconstruction and morphometry of newborn whole femurs from BSP+/+ (+/+) and −/− newborn mice. (B) Histomorphometry of trabecular bone in newborn femurs from +/+ and −/− mice. Data are Mean±SEM of N = 4 to 10 mice; *:p<0.05, **:p<0.01 Vs +/+, Mann-Whitney U Test. (C) Micrographs of Goldner's trichrome and Von Kossa staining of BSP+/+ and BSP−/− newborn femur sagittal sections. Yellow polygon: ROI for histomorphometry of the trabecular bone. Bar  = 400 µm.

Microtomographic analysis of femurs in newborn BSP+/+ and BSP−/− mice showed that mutant bones had a lower global bone volume, explained by a thinner cortical envelope (see also pictures in Fig. 5C) as well as a lower tissue mineral density (Fig. 5A), as previously reported [6]. In contrast, histomorphometric analysis of femoral trabecular bone showed no difference in BV/TV between mutant and wild type (Fig. 5B, C). Osteoclast parameters, Oc.S/BS and Oc.N/B.Ar did not differ between newborn BSP−/− and BSP+/+ mice (Fig. 5B).

### Expression of factors regulating long bone growth is altered in BSP−/− mice

**Figure 6:**
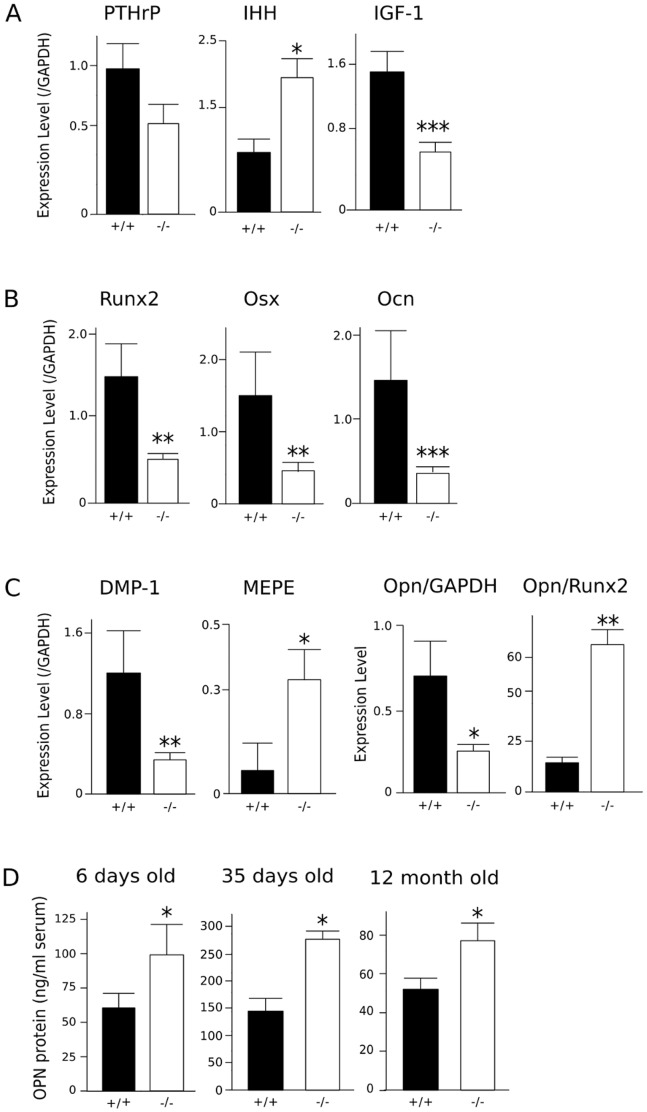
Growth plate and osteoblast marker protein expression and blood levels in newborn and adult BSP+/+ and BSP−/− mice. QRT-PCR was performed 6 days after birth on collected whole femurs and tibias including the growth plates, ground and extracted in Tri-reagent. Messenger RNA expression of growth plate regulators IHH, PTHrP and IGF-1 (A), markers of bone formation Runx2, Osx and Ocn (B) and SIBLING proteins DMP1, MEPE and Opn (C) were assessed in samples from BSP+/+ (N = 5) and BSP−/− (N = 9) mice. Expression levels are normalized on the housekeeping gene GAPDH, and on Runx2 levels for Opn (C). ELISA assay of Opn (D) in the serum of 6 days (N = 5 pools), 35 days and 12 month old (N = 5 male) BSP+/+ and BSP−/− mice. Data are Mean±SEM; *: p<0.05, **: p<0.01, ***: p<0.001 vs BSP+/+, Mann-Whitney U test.

QRT-PCR analysis of 6 days old mouse bones showed that PTHrP expression was decreased, albeit not significantly (P = 0.11) in 6 days old BSP−/− long bones, and IHH expression was increased (Fig. 6A). Bone expression of IGF-1, a major growth-promoting signal for skeletal development, was decreased in mutant bones (Fig. 6A).

### BSP−/− mice display a decrease in bone formation markers and higher levels of circulating osteopontin

In 6 days old pups, the expression of early osteoblast markers, Runx2 and Osx was decreased as well as that of the late marker Ocn (Fig. 6B) and the SIBLING DMP1 (Fig. 6C). Interestingly, the expression of the other SIBLING protein MEPE was found to be increased in BSP−/− long bones (Fig. 6C), while expression of Opn was decreased when normalized on GAPDH but was 5 fold increased when normalized on Runx2 (Fig 6C). ELISA assay of Opn in the blood of 6 day old mice showed significantly higher values (+64%) in BSP−/− than in the BSP+/+, and interestingly they were still higher (+48%) in the blood of aged, 12 month old mutant mice whose mineral density is no longer different from the wild type ([6], Fig. 6D).

### Progressive development of the adults BSP−/− bone phenotype

**Figure 7:**
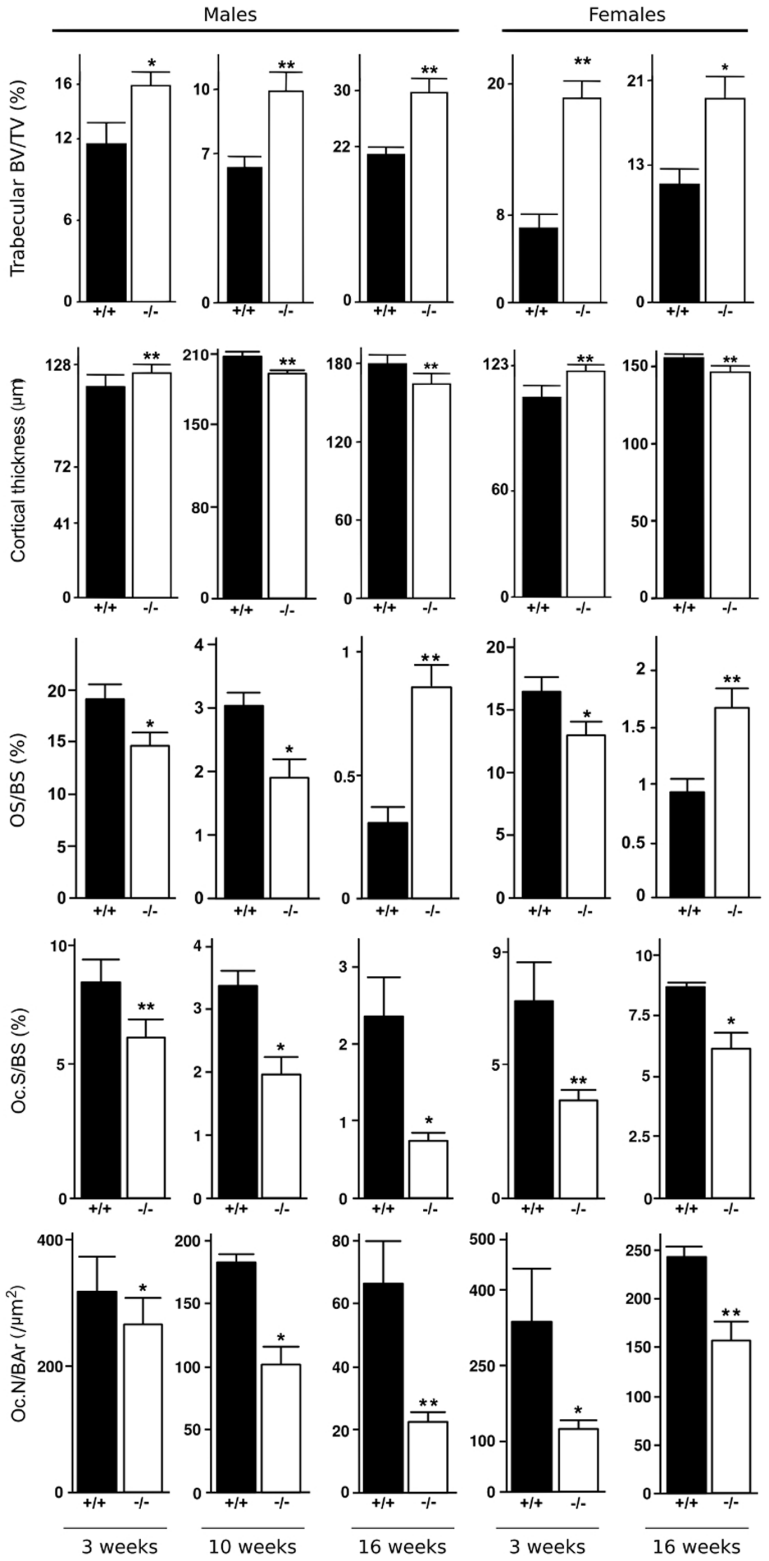
Static histomorphometric parameters and cellular activities in femur bones from 3, 10 and 16 week old BSP+/+ (+/+) and BSP−/− (−/−) mice. Data are Mean±SEM of N = 6 to 10 mice; *:p<0.05, **:p<0.001 vs BSP+/+, Mann-Whitney U Test.

As early as 3 weeks after birth, and at 10 and 16 weeks of age trabecular BV/TV in the femurs of BSP−/− mice was higher than in BSP+/+, in both male and female (Fig. 7). This was concomitant with a decrease of osteoclast parameters, both Oc.S/BS and Oc.N/B.Ar in mutant mice of both sexes. Cortical bone of BSP−/− mice became transiently thicker at 3 weeks respective to BSP+/+, to become thinner at 10 and 16 weeks of age (Fig. 7). Interestingly, OS/BS values were significantly lower in BSP−/− mice at 3 and 10 weeks of age, to become higher than in BSP+/+ at 16 weeks (Fig. 7), as previously published [6]. The impact of the BSP−/− mutation on post-natal skeletal development is summarized in Table 2.

**Table 2:** Impact of the absence of BSP on postnatal development of the skeleton.

Age	Growth plate	Cortical bone	Trabecular bone	Osteoclasts	Osteoid
Newborn	GP/HZ < +/+	< +/+	≈ +/+	≈ +/+	-
3 weeks	HZ > +/+, PZ < +/+	> +/+	> +/+	< +/+	< +/+
10 weeks	GP/HZ ≈ +/+	< +/+	> +/+	< +/+	< +/+
16 weeks	GP ≈ +/+, ∼ inactive	< +/+	> +/+	< +/+	> +/+

The table compares to wild type (+/+), at different ages of developing mutant mouse long bones, the whole growth plate (GP), hypertrophic (HZ) and proliferating zone (PZ) thickness, cortical bone thickness, trabecular bone volume (BV/TV), osteoclast surface/number and osteoid thickness.

## Discussion

In this study, we show that mice lacking BSP are smaller than their wild type counterparts since birth and throughout their developmental process. The study of their skeleton did not show any other morphological effect of the mutation. However, the amount of primary mineralized bone is lower in the skull and long bone cortex of BSP−/− newborn mice. Moreover, and as previously shown [6], the volumetric bone density in BSP−/− newborns is lower than in BSP+/+.

Our study was further complicated by the unexpected discovery that weaning female BSP−/− mice do not display normal care for their pups. That the lack of BSP, an extracellular matrix protein would affect behavior is both surprising and novel. To the best of our knowledge, the only other case of a behavioral impact of the absence of a SIBLING member is the "circling behavior" and hyper-reactivity to touch of DMP1−/− mice [12]. This has been ascribed to the under-mineralization of the vestibular bone, which would alter sensory (balance) perception in this line [12]. In a recent analysis of the genetics of nest building in two inbred mouse strains, chromosome 5 -which carries the SIBLING locus in mice- displayed no direct effect QTL, but was found involved in several epistatic effects [13]. Extensive studies, including ethological, will be required to assess the full impact of the BSP−/− mutation on mouse behavior and evaluate whether it reflects a direct (e.g. neurological) effect or an indirect, e.g. sensory one as for the DMP1 knockout. Because we kept the mutants as a separate line, and the mother's aberrant behavior might have a thermic and/or trophic impact on the pup's development, we conducted a thorough analysis of the growth of BSP+/+, +/− and −/− mice from mothers of various genotype. Of note, variations in growth kinetics were observed according to the number of pups (not shown) as was expected [14], and we took care to compare genotype effects between litters of similar sizes. The results (Fig. 3) show without doubt that the phenotype of the mutant mice only reflects the absence of the BSP protein, and is thus independent from the mother's genetics.

BSP is expressed in hypertrophic chondrocytes [15], and given its effects on endothelial cells migration on the one hand [16] and hydroxyapatite nucleation on the other hand [17], one could expect an effect of the lack of BSP on the onset of endochondral ossification. Surprisingly, this process does not seem to be affected in BSP−/− mice, as no delay appears in the onset of ossification centers, and trabecular BV/TV and osteoclast parameters in newborn femurs are similar between genotypes. This suggests that some compensatory mechanisms may exist to override the absence of BSP. In particular, other SIBLING proteins are expressed by hypertrophic chondrocytes and osteoblasts in the chondro-osseous border, and are thought to play an important role in endochondral bone ossification [18] (see below). Further analyzes are needed to determine the exact role of BSP in the onset of endochondral ossification and the possible compensatory effects of other SIBLING members.

Nonetheless, newborn BSP−/− femurs display thinner growth plates, indicating that the long bone growth kinetics is already altered at that stage, as also witnessed by the slightly smaller size and lighter weight of newborn mutant mice. Bone growth is achieved by proliferation and hypertrophy of chondrocytes, then removal of the cartilage template by osteoclasts and its replacement with woven bone by osteoblasts. This process is subject to a regulation loop involving PTHrP secreted by perichondrial cells and resting chondrocytes, which promotes chondrocyte proliferation and inhibits their final differentiation, and IHH secreted by resting and hypertrophic cells, which stimulates PTHrP expression as a functional negative feedback [19]. Our QRT-PCR analysis in early post-natal (6 days) mice found that IHH expression was surprisingly increased in the BSP−/−, despite the thinner hypertrophic zone, while PTHrP actually showed a trend to reduction, suggesting a disruption of the feedback loop. Most interestingly, the expression of IGF-1, a major factor inducing both chondrocyte proliferation and hypertrophy, was found to be severely reduced in BSP−/− post-natal long bone. While the interplay between circulating and local, autocrine/paracrine IGF-1 is complex (see [20] for a recent review), it is known that tissue IGF-1 is crucial for post natal bone growth, and that only a severe reduction of circulating IGF-1 (down to ∼15% of normal) significantly affects mouse long bone size [21]. The reduction of IGF-1 expression in BSP−/− mice might thus be a major factor explaining the slower growth of mutant skeleton. However, the IGF-1 pathway and the IHH/PTHrP pathway have been shown to act independently [22], suggesting a more complex picture. That both higher IHH and lower IGF-1 expression would reflect phenotypic alteration of hypertrophic chondrocytes, the only stage which has been shown to express BSP, is one hypothesis to be tested. Of note, our PCR analysis concerned whole bones, and therefore the results did not distinguish between chondrocyte and osteoblast contribution, e.g. to IGF-1 expression. IGF-1 is also a major factor regulating bone formation activity, and reduction/absence of IGF-1 signaling results in osteopenia [20], a phenotype observed in growing BSP−/− mice (see below).

The proliferative zone was found to be thinner at 3 weeks in BSP−/− than in BSP+/+ mice, suggesting that chondrocyte proliferation/survival is altered in mutants perhaps through a cross-talk with hypertrophic chondrocytes. The total width of the growth plate is not altered, as the hypertrophic zone is thicker in 3 week old BSP−/− mice. This may result at least in part from the reduced osteoclast numbers and thus activity at this stage in the absence of BSP [7], which could impair the proper resorption of the cartilage template. Further studies will be needed to unravel the multiple mechanisms through which the absence of BSP affects the dynamic of bone growth.

The lower bone mass observed in newborn BSP−/− mice is congruent with the QRT-PCR results showing lower expression of both early (Runx2, Osx) and late (Ocn, DMP1) bone formation markers, as well as the lower levels of IGF-1 expression. We also confirmed in this study the lower mineral density of newborn BSP−/− bone matrix, extending the observations to developing digits and calvariae (Fig. 2). The higher level of MEPE expression observed in post-natal mutant long bones is intriguing, as this SIBLING protein, in particular through its cleavage releasing an "Acidic Serine-Aspartate Rich MEPE associated" (ASARM) peptide, is a potent inhibitor of matrix mineralization in bone [23] but also in the growth plate [24]. Of note, levels of MEPE expression in adult (2 month old) BSP−/− bones were not found to differ from wild type mice [25]. Opn is also a major inhibitor of bone mineralization, as appears from the hyper-mineralized Opn−/− mouse bones [26], as well as the rescue of bone hypo-mineralization induced by tissue non-specific alkaline phosphatase (*Akp2*) gene knockout when these mice are crossed with the Opn−/− [27]. Surprisingly, Opn expression was found to be lower in mutant long bones at day 6, while serum levels were higher than in wild type. However, osteoblasts are a major source of Opn in bone, and when normalized to Runx2, a marker of the osteoblast/chondrocyte lineage Opn levels were indeed higher in BSP−/− mice, suggesting a relative over-expression of the protein. Thus, the under-mineralized matrix observed in young mice in the absence of BSP might reflect at least in part the action of MEPE and Opn. Of note, mineralization levels progressively equalize in aging BSP+/+ and −/− mice [6], which we interpret as a main action of BSP on primary mineralization, in congruence with the higher osteoid surfaces observed in adult long bones (see below). Thus, the permanence of higher serum Opn levels in aging BSP−/− mice (Fig. 6D) suggests that its action might not be dominant in this model, or at least not at all stages of skeletal life. Multiple knockouts of SIBLING genes would clearly be helpful to unravel such questions.

Bone growth in rodent is continuous, but its rate decreases with age, along with growth plate activity, which we found to be reduced at 10 weeks of age in the 129 sv/CD1 mice, and nearly arrested at 16 weeks (Fig. 4). As the BSP−/− mice grow, an accumulation of trabecular bone is seen as early as 3 weeks after birth, in concert with a reduction of osteoclast numbers and surfaces, suggesting that the increase in trabecular BV/TV after birth results at least in part from defective resorption. Lower OS/BS are observed in 3 week and 10 week old BSP−/− mice, likely reflecting lower bone forming activity, as previously shown at the peak of repair bone deposition in cortical defect repair of BSP−/− mice [28]. With the reduction of growth rate, the modeling process, in which formation and resorption are uncoupled slows down, and primary woven bone is gradually replaced with lamellar bone, by the process of bone remodeling. While secondary ossification progressively supersedes primary ossification, the overall cellular activity is reduced. As previously shown [6], in the less active, mostly remodeling trabecular bone of 16 week old BSP−/− mice, OS/BS values are higher than in wild type, likely reflecting delayed primary mineralization. The thinner cortical bones observed in mutant mice at 10 and 16 weeks of age reflect lower periosteal and endosteal BFR, as documented elsewhere [25]. The transient thickening of the cortical bone in 3 week old BSP−/− mice is intriguing, and may hint at a temporary imbalance of the modeling process during the post-natal crisis period, marked by a drop in osteoclast numbers. Clearly, a closer analysis has to be done of the very early times of BSP−/− growth to assess these hypothesis.

In conclusion, we confirmed in this study that BSP plays an important role in long bone growth kinetics, as well as membranous/cortical primary bone formation and mineralization. Endochondral development is however normal in BSP−/− mice and the accumulation of trabecular bone observed in the adults develops progressively in the weeks following birth. The effects of the absence of BSP on chondrocyte proliferation, differentiation and activity, the compensatory mechanisms that allow normal endochondral bone development in BSP−/− mice as well as the role played by IGF-1, MEPE and Opn in the absence of BSP will have to be explored.
